# Supplementary material for: Causal interplay between lactose intolerance and gut microbiota: a combined bidirectional Mendelian randomization and in vivo validation study
Source: Front Nutr. 2026 Jun 1;13:1803337. doi: 10.3389/fnut.2026.1803337 (PMC13265576; doi:10.3389/fnut.2026.1803337)
Supplement: Supplementary file 1 [file Data_Sheet_1.zip › supplementary materials/Forward/forest plot/ebi-a-GCST90027771.finngen_R12_E4_LACTONAS.pdf]

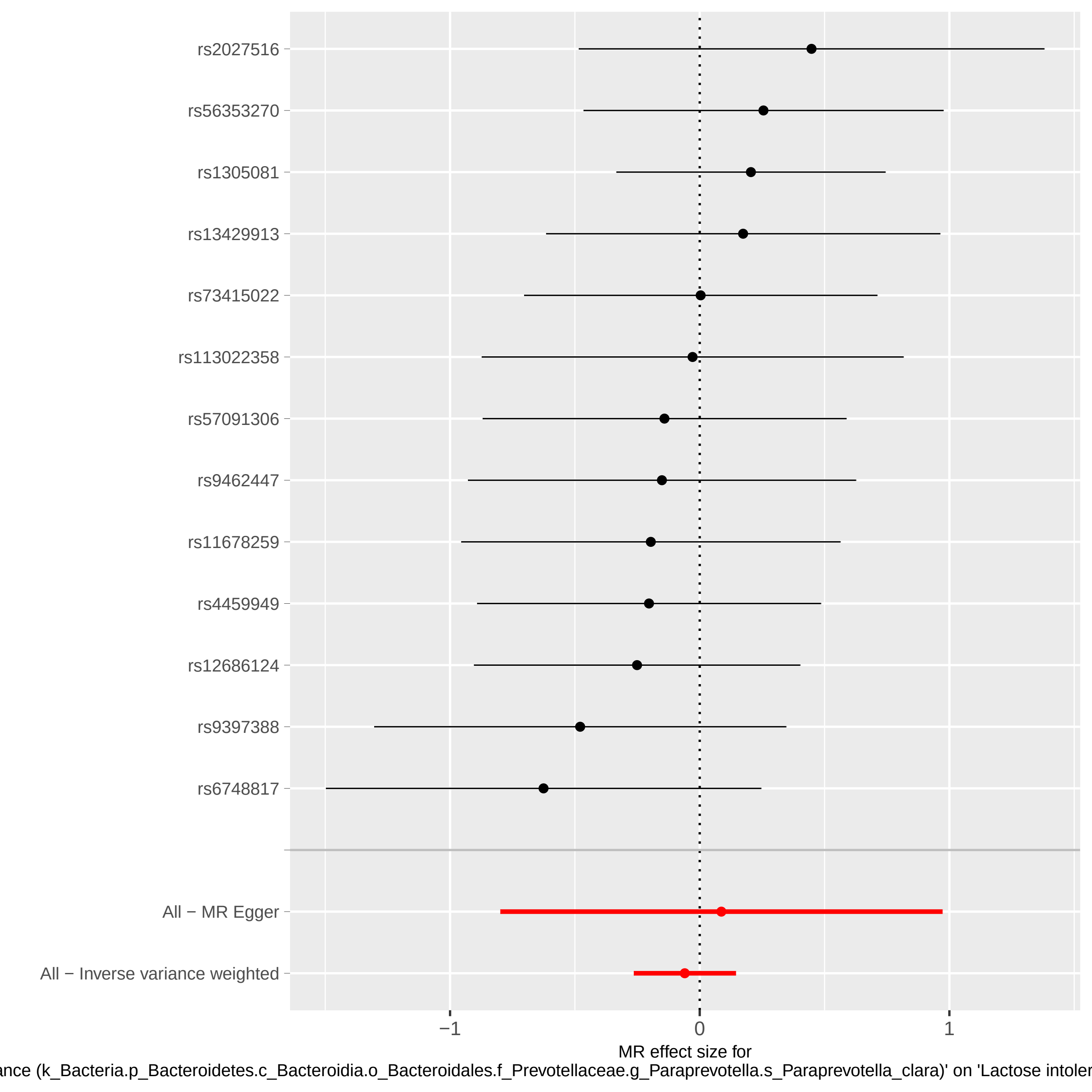

rs2027516
rs56353270
rs1305081
rs13429913
rs73415022
rs113022358
rs57091306
rs9462447
rs11678259
rs4459949
rs12686124
rs9397388
rs6748817
All − MR Egger
All − Inverse variance weighted
−1
0
1
MR effect size for
ance (k_Bacteria.p_Bacteroidetes.c_Bacteroidia.o_Bacteroidales.f_Prevotellaceae.g_Paraprevotella.s_Paraprevotella_clara)' on 'Lactose intoler
